# Biomechanical properties of a novel nonfusion artificial vertebral body for anterior lumbar vertebra resection and internal fixation

**DOI:** 10.1038/s41598-021-82086-7

**Published:** 2021-01-29

**Authors:** Jiantao Liu, Xijing He, Binbin Niu, Yin Yang, Yanzheng Gao, Jintao Xiu, Hongbo Wang, Yanbiao Wang

**Affiliations:** 1 Department of Orthopedics, The First Affiliated Hospital of Xi’an Jiaotong University, Xi’an, 710061 China; 2 Department of Orthopedics, Second Affiliated Hospital of Xi’an Jiaotong University, Xi’an, 710004 China; 3 Department of Orthopedics, Second Affiliated Hospital of Xi’an Medical University, Xi’an, 710021 China; 4 Department of Orthopedics, Xi’an Central Hospital, Xi’an, 710003 China; 5 Department of Spine and Spinal Cord Surgery, Henan Provincial People’s Hospital, People’s Hospital of Zhengzhou Umiversity, Zhengzhou, 450003 China

**Keywords:** Medical research, Engineering, Mathematics and computing

## Abstract

The aim of the study was to evaluate the biomechanical properties of a novel nonfused artificial vertebral body in treating lumbar diseases and to compare with those of the fusion artificial vertebral body. An intact finite element model of the L1–L5 lumbar spine was constructed and validated. Then, the finite element models of the fusion group and nonfusion group were constructed by replacing the L3 vertebral body and adjacent intervertebral discs with prostheses. For all finite element models, an axial preload of 500 N and another 10 N m imposed on the superior surface of L1. The range of motion and stress peaks in the adjacent discs, endplates, and facet joints were compared among the three groups. The ranges of motion of the L1–2 and L4–5 discs in flexion, extension, left lateral bending, right lateral bending, left rotation and right rotation were greater in the fusion group than those in the intact group and nonfusion group. The fusion group induced the greatest stress peaks in the adjacent discs and adjacent facet joints compared to the intact group and nonfusion group. The nonfused artificial vertebral body could better retain mobility of the surgical site after implantation (3.6°–8.7°), avoid increased mobility and stress of the adjacent discs and facet joints.

## Introduction

Lumbar fractures, tumors, infections and other diseases seriously threaten human health^1,2^. Improper treatment can easily lead to spinal deformity, paralysis, back pain and other complications, seriously reducing patient quality of life. Since Hamdi et al. successfully performed vertebral body replacement in two patients with spinal tumors^3^, vertebral body resection and fusion has become a classic surgical method for treating the above mentioned diseases^4^. Although the treatment method can achieve complete spinal decompression and restore the height and stability of the lumbar spine^5,6^, the operation often requires the fusion of three or more vertebrae, which inevitably leads to loss of the physiological and motor function of the spine in the surgical area. Some studies have reported that the pressure on the adjacent intervertebral disc and articular process will increase after fusion, thus accelerating the degeneration of the adjacent segment^7^. In severe cases, another operation is required. To address the above limitations, many scholars have focused the development of nonfusion prostheses for the spine, thus promoting the development and application of an artificial disc, elastic rod, artificial nucleus pulposus and other prostheses. A large number of clinical studies have shown that an artificial disc and nucleus pulposus have achieved satisfactory results in restoring intervertebral space height and retaining spinal motor function^8,9^. However, due to the limitations of their own structures, the above prostheses cannot reconstruct the vertebral height and thus are difficult to use in patients with vertebral resection. In view of this, developing a new prosthesis suitable for the lumbar spine that can not only retain the physiological and motor function of the surgical area but also reconstruct the height and stability of the lumbar vertebra is of great clinical significance. According to the anatomical characteristics of the human lumbar spine, we developed a novel lumbar implant, the movable artificial lumbar vertebra (MALV), and obtained a national invention patent (No. ZL201610285603. X) and international Patent Cooperation Treaty (PCT) patent (No. PCT/CN2016/104,550). We tested the mobility and stability of the novel prosthesis through in vitro mechanical tests with human specimens, and the results showed that the novel prosthesis could not only replace vertebral lesions but also reconstruct the stability and mobility of the surgical site^10,11^. Although we found that the new prosthesis could reduce the activity of the adjacent intervertebral space compared with the fused artificial vertebral body, the stress effect on the surrounding tissues needed further study. Therefore, finite element (FE) analysis was used to study the biomechanical properties of this new prosthesis to provide a reference for its long-term biomechanical safety after implantation.

## Results

### Model validation

**Figure 1:**
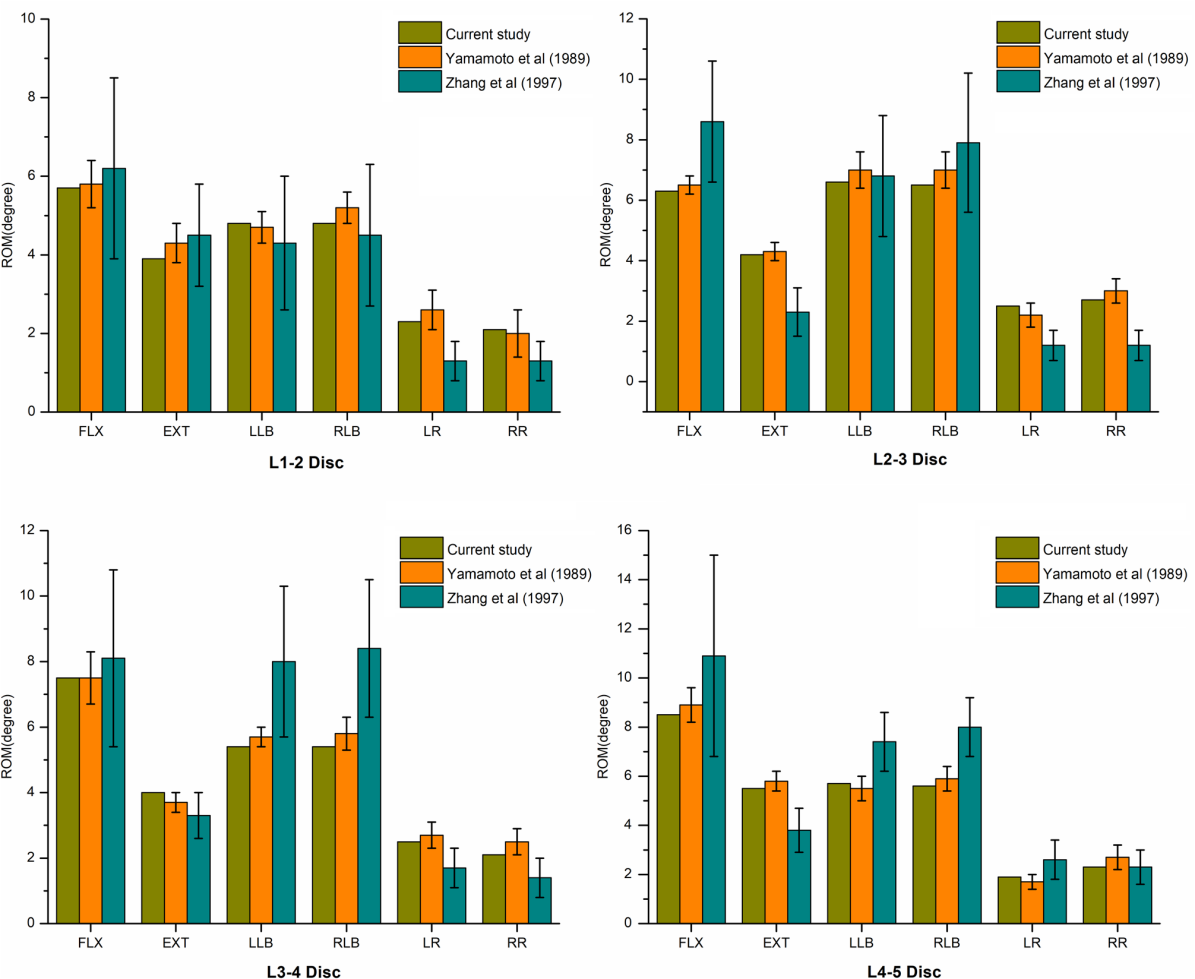
Comparison of ROMs between the current intact FE model and the outcomes of previous studies.

The ROMs of L1-L2 in the intact group in flexion, extension, left lateral bending, right lateral bending, left rotation and right rotation were 5.7°, 3.9°, 4.8°, 4.8°, 2.3° and 2.1°, respectively. The corresponding ROMs of L2-L3 were 6.3°, 4.2°, 6.6°, 6.5°, 2.5° and 2.7°, while those of L3-4 were 7.5°, 4.0°, 5.4°, 5.4°, 2.5° and 2.1°. The L4-5 ROMs in flexion, extension, left lateral bending, right lateral bending, left rotation and right rotation were 8.5°, 5.5°, 5.7°, 5.6°, 1.9° and 2.3°, respectively. The results of the ROMs were in accordance with the findings of previous cadaveric studies^12,13^ (Fig. 1), suggesting that the intact L1–L5 FE model in the present study was successfully constructed and could be used for further analysis.

### Disc ROM

**Figure 2:**
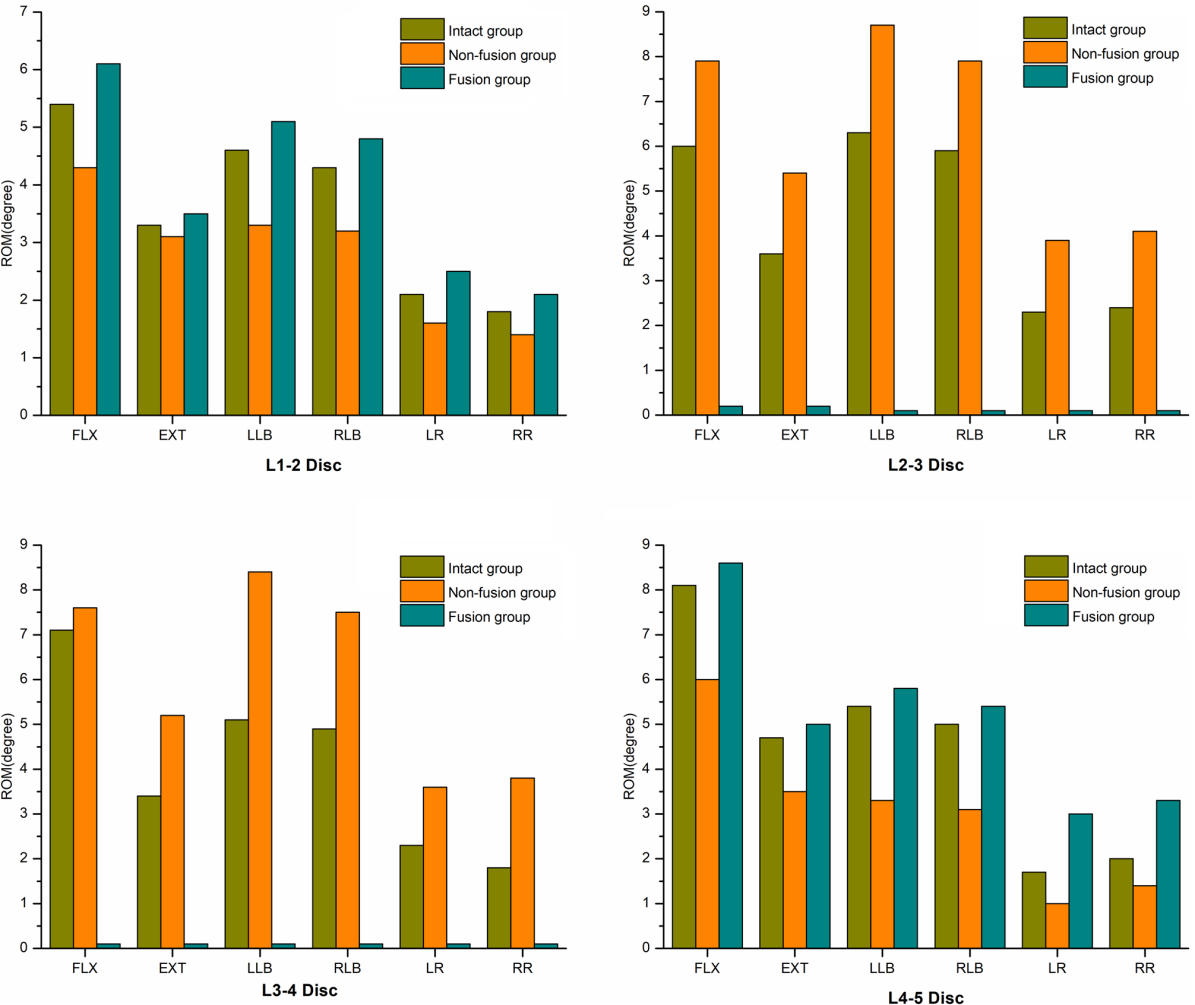
Comparison of disc ROMs among different groups in flexion, extension, left lateral bending, right lateral bending, left rotation and right rotation.

The ROMs of the L1–2, L2–3, L3–4 and L4–5 discs in flexion, extension, left lateral bending, right lateral bending, left rotation and right rotation among the different groups are shown in Fig. 2. The ROMs of the discs adjacent to the surgical site (L1–2 and L4–5) in all the above directions were obviously greater in the fusion group than in the intact group. However, the ROMs of the discs in the surgical site (L2–3 and L3–4) were significantly decreased in the fusion group compared with those in the intact group. The ROMs of the discs in the nonfusion group showed an opposite trend to those in the fusion group. The ROMs of the L1–2 and L4–5 discs in flexion, extension, left lateral bending, right lateral bending, left rotation and right rotation were obviously decreased and the L2–3 and L3–4 ROMs were significantly increased in the nonfusion group compared with those in the intact group.

### Adjacent disc stress

**Figure 3:**
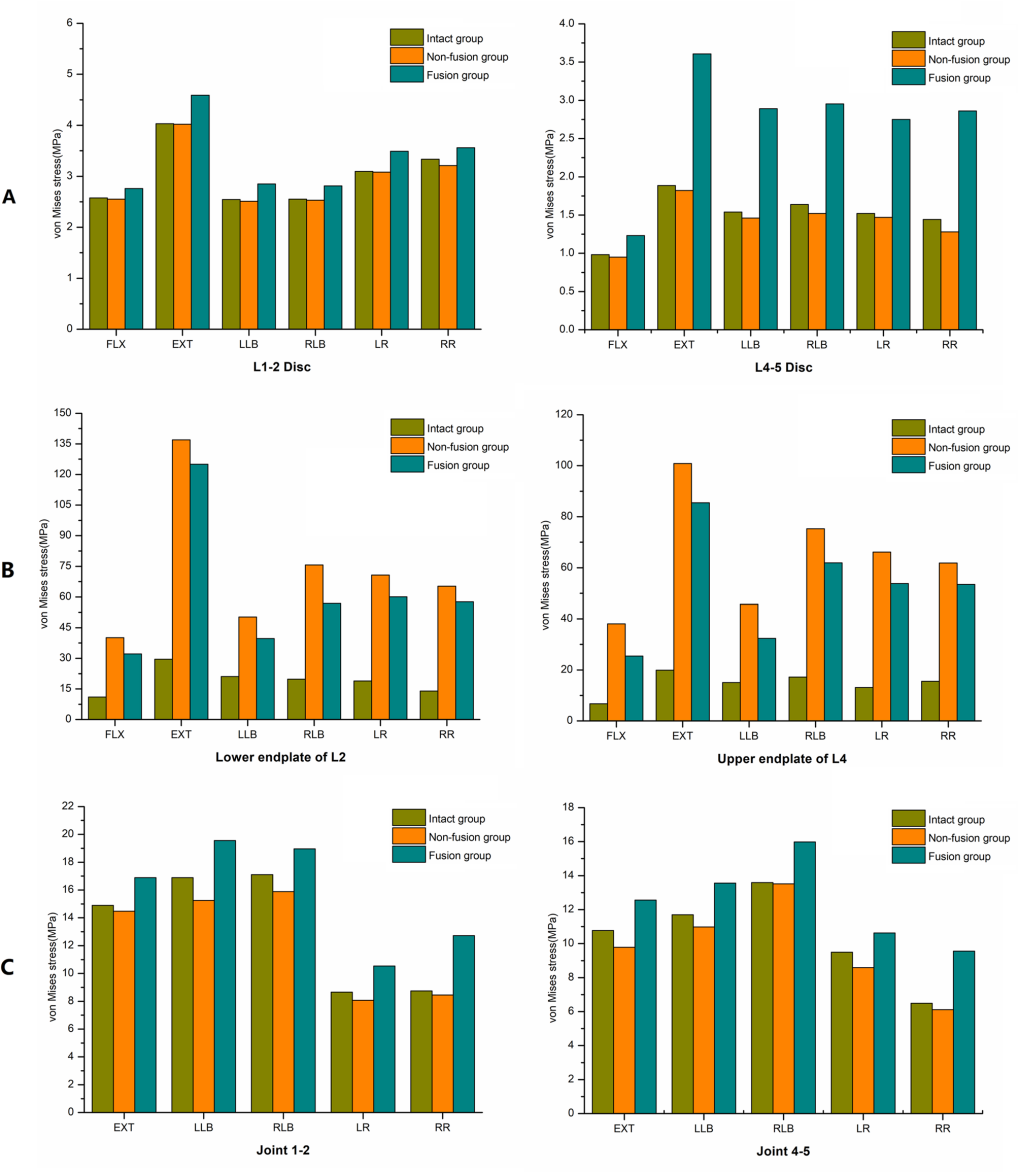
Comparisons of von Mises stress peaks: (**A**) adjacent disc stress, (**B**) endplate stress, (**C**) facet joint stress.

The von Mises stress peaks of the adjacent discs (L1–2 and L4–5) are shown in Fig. 3A. The maximum von Mises stresses of the L1–2 and L4–5 discs in the fusion group in flexion, extension, left lateral bending, right lateral bending, left rotation and right rotation were 2.76 MPa, 4.59 MPa, 2.85 MPa, 2.81 MPa, 3.49 MPa, and 3.56 MPa and 1.23 MPa, 3.61 MPa, 2.89 MPa, 2.95 MPa, 2.75 MPa, and 2.86 MPa, respectively, which were significantly greater than those in the intact group. The ranges of maximum von Mises stresses of the L1-2 and L4-5 discs in the above directions were 2.55–4.02 MPa and 0.95–1.82 MPa, respectively, in the non-fusion group, which were slightly lower than those in the intact group (2.55–4.03 MPa and 0.98–1.8 MPa, respectively).

### Endplate stress

The von Mises stress peaks and the stress distributions of the adjacent endplates (lower endplate of L2 and upper endplate of L4) are shown in Fig. 3B. The maximum von Mises stresses of the lower L2 endplate in flexion, extension, left lateral bending, right lateral bending, left rotation and right rotation in the intact group were 11.04 MPa, 29.55 MPa,21.07 MPa, 19.76 MPa, 18.92 MPa and 13.96 MPa, respectively, which were significantly lower than those in the fusion group (32.20 MPa, 125.07 MPa, 39.74 MPa, 56.89 MPa, 60.06 MPa, and 57.66 MPa, respectively) and the nonfusion group (40.11 MPa, 136.97 MPa, 50.26 MPa, 75.64 MPa, 70.69 MPa, and 65.23 MPa, respectively). The corresponding maximum von Mises stresses of the upper L4 endplate in the nonfusion group were 38.10–100.85 MPa, which were obviously greater than those in the intact group (6.76–19.90 MPa) and the fusion group (25.49–85.50 MPa).

### Facet joint stress

**Figure 4:**
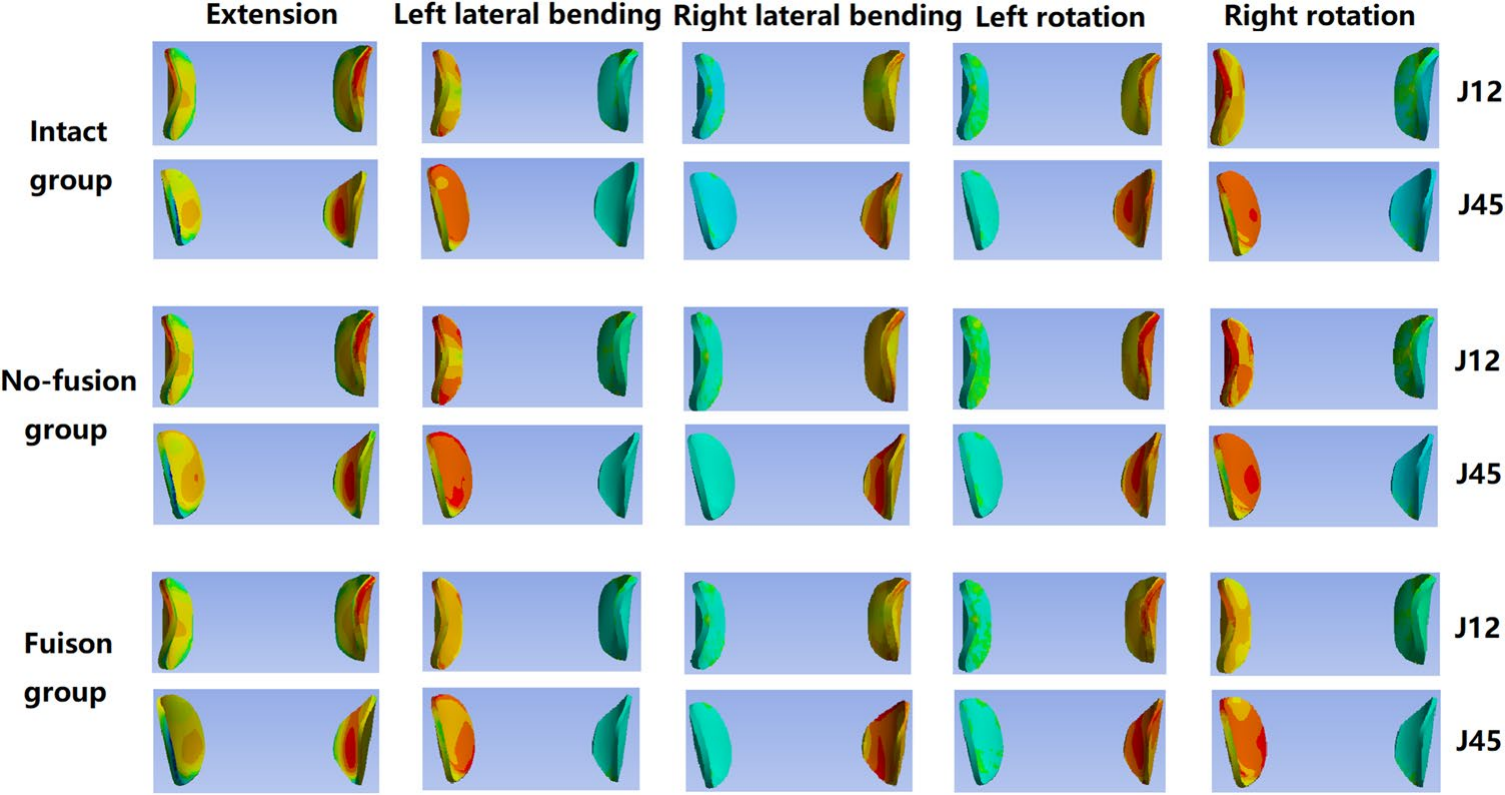
The von Mises stress distributions of the adjacent facet joints.

The von Mises stress peaks and the stress distributions of the adjacent facet joints (J12 and J45) are shown in Figs. 3C and 4, respectively. The maximum von Mises stresses of J12 in extension, left lateral bending, right lateral bending, left rotation and right rotation in the nonfusion group were 14.48 MPa, 15.25 MPa, 15.89 MPa, 8.07 MPa and 8.45 MPa, respectively, which were similar with those in the intact group (14.89 MPa, 16.89 MPa, 17.11 MPa, 8.65 MPa, and 8.75 MPa, respectively) but significantly lower than those in the fusion group (16.89 MPa,19.56 MPa, 18.96 MPa, 10.54 MPa, and 12.72 MPa, respectively). The corresponding maximum von Mises stresses of J45 in the nonfusion group were 6.12–13.52 MPa, which were obviously lower than those in the fusion group (9.56–15.98 MPa) but similar with those in the intact group (6.48–13.59 MPa).

## Discussion

Anterior lumbar subtotal resection is widely used to treat lumbar tuberculosis, fracture, tumors and other diseases and often uses titanium cages combined with titanium plates or screw rods. Although this operation can achieve full decompression of the spinal cord and reconstruct the height and stability of the vertebra under direct vision, it often requires the fusion of three or more vertebrae, resulting in loss of mobility in the surgical area. Long-term follow-up results showed that the incidence of complications such as adjacent disc degeneration was significantly increased after this procedure^14^. The appearance of artificial intervertebral discs changed the fusion concept and integrated the concept of mobility into the spinal field. The long-term follow-up results showed that the artificial intervertebral disc had a significant effect on recovery of the height of the intervertebral space and retention of spinal motor function. However, artificial intervertebral discs are difficult to use in patients with vertebrotomy due to their inherent structural limitations. Therefore, there is an urgent need to design a new type of prosthesis that can not only preserve the mobility of the surgical site but also restore the height and stability of the lesion site. In the early stage, a new type of lumbar prosthesis was designed according to the anatomical parameters of lumbar vertebrae – the MALV. We tested the mobility and stability of the MALV with an in vitro mechanics test^11^, and the results showed that the new prosthesis could not only replace the vertebral body of the lesion but also reconstruct the stability and mobility of the surgical site. However, the stress effect of the new prosthesis on the surrounding tissues after implantation still needed to be further studied.

The FE method is a kind of discrete method in numerical calculations and is based on the matrix method in structural mechanics, elastic mechanics and other fields. Belytschko et al.^15^ first applied FE analysis to the study of spine biomechanics in 1973. Subsequently, many scholars verified the reliability of the stress and strain results of FE analysis through relevant experiments and obtained relatively positive results^16–20^. In this study, a FE model of the whole lumbar spine was established in the intact group using the CT data of a healthy adult and validated using published experimental data. On this basis, FE models of the fusion and nonfusion groups were successfully established. The ROMs were evaluated in the intact model and models with prostheses. The stresses exerted on the adjacent discs, endplates and facet joints were analyzed.

According to our results, the ROMs of the L2–3 and L3–4 intervertebral discs in the fusion group were significantly lower than those in the intact group in the directions of flexion, extension, lateral bending and rotation, while the ROMs of the L2–3 and L3–4 intervertebral discs in the nonfusion group were higher than those in the intact group. In the fusion group, the ROMs of the adjacent discs (L1–2 disc and L4–5 disc) in the above directions were significantly increased compared with those in the intact group and nonfusion group, while the ROMs of the adjacent discs in the nonfusion group in the flexion, extension, lateral bending and rotation directions were decreased compared with those in the intact group. This is consistent with our previous results from in vitro mechanics tests on fresh cadaver specimens^11^. It was suggested that fusion surgery would seriously affect the physiological motor function of the lumbar spine at the surgical site and increase the intervertebral disc ROM in the adjacent segments, which was consistent with the previous research results of Biswas, JK et al^21^. Although the ROMs of the adjacent discs to the surgical site were reduced in the nonfusion group compared with those in the intact group, the difference between the two groups was not obvious. Therefore, after implantation of the new prosthesis, not only can the mobility of the surgical site be better retained but the increased mobility of the adjacent discs caused by fusion can also be avoided to some extent.

The maximum von Mises stresses of the adjacent discs (L1–2 disc and L4–5 disc) and facet joints (J12 and J45) in the fusion group in the directions of flexion, extension, lateral bending and rotation were significantly increased compared with those in the intact group and the nonfusion group, while the maximum von Mises stresses of the adjacent discs (L1–2 disc and L4–5 disc) and facet joints (J12 and J45) in the nonfusion group were decreased compared with those in the intact group. Thus, to some extent, the stress in the adjacent segments will increase after fusion, leading to accelerated degeneration, which is consistent with the increased stress in the adjacent segments reported by Kim H J et al. after fusion. After implantation, the new prosthesis can effectively avoid this increase in stress in the adjacent segments after surgery to effectively reduce the incidence of adjacent segment degeneration.

The results of the maximum von Mises stresses on the endplate of the surgical site (lower endplate of L2 and upper endplate of L4) showed that the maximum von Mises stresses in the fusion group and nonfusion group in the flexion, extension, lateral bending and rotation directions were significantly higher than those in the intact group. The reason is that the intervertebral discs in the intact group have a certain elastic cushioning, which can reduce the stress to the adjacent endplates to a certain extent. However, the prostheses in the fusion group and nonfusion group were made of metal materials, which lack an elastic buffer, resulting in a significant stress increase in the adjacent endplate. However, the maximum von Mises stresses of the adjacent endplates in the above directions in the nonfusion group were much higher than those in the fusion group. Due to the use of titanium plate or pin rod auxiliary fixation, the load of the titanium cage can be shared to a certain extent, reducing the stress in the contact area between the titanium cage and the end plate. Therefore, to avoid the high sinking rate after traditional titanium cage implantation, the contact area between the new prosthesis and the endplate was carefully designed to not only increase the contact area between the new prosthesis and the endplate but also match the anatomical morphology of the adjacent endplate.

In conclusion, this study confirmed that this new type of lumbar prosthesis could better retain the mobility of the surgical site after implantation, avoid increases in mobility and stress of the adjacent discs and stress of the facet joints, and reduce the incidence of adjacent segment degeneration after long-term implantation. However, the long-term stability, fatigue resistance, wear resistance and other mechanical properties of the new prosthesis after implantation still need to be further studied.

## Materials and methods

### Development of MALV

**Figure 5:**
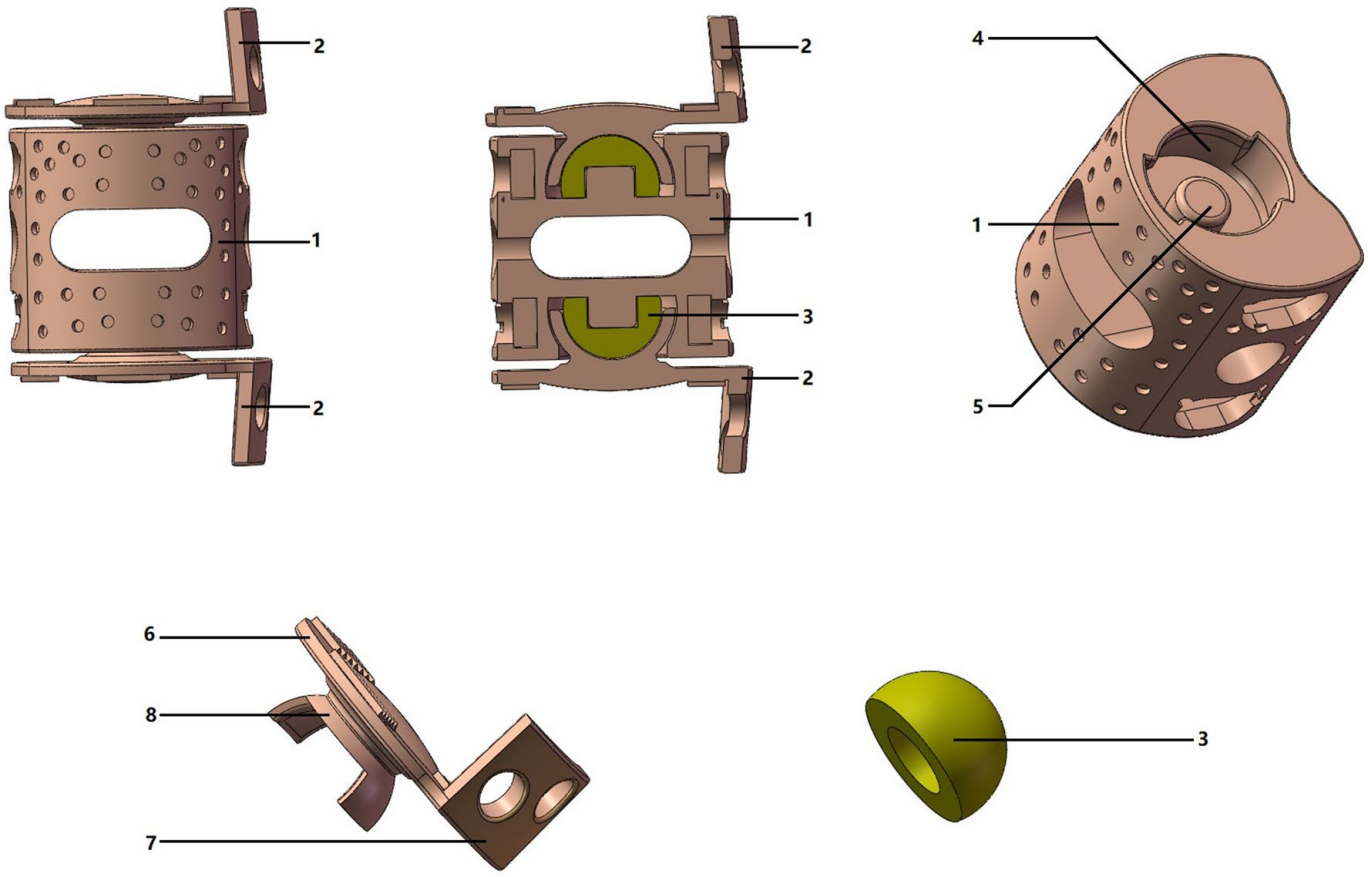
Three-dimensional model of the MALV: 1 vertebral body part, 2 intervertebral disc part, 3 composite material ball, 4 grooves on the upper and lower ends, 5 a fixed column in the center, 6 sunken back plate, 7 curved side plate, and 8 the spherical shell structure.

The MALV prosthesis consists of three parts: vertebral body part, intervertebral disc part and composite material ball (Fig. 5). The vertebral body part is an irregular cylinder with a depression in the back, grooves on the upper and lower ends and a fixed column in the center. The intervertebral disc part consists of a sunken back plate, curved side plate, and cylindrical protuberances with ends connected to the spherical shell structure. The composite material ball is composed of Ultrahigh molecular weight polyethylene (UHMWPE) that forms a ball and socket joint with a spherical shell structure. The vertebral body part and intervertebral disc part were made of Ti6Al4V.

### FE modeling

**Figure 6:**
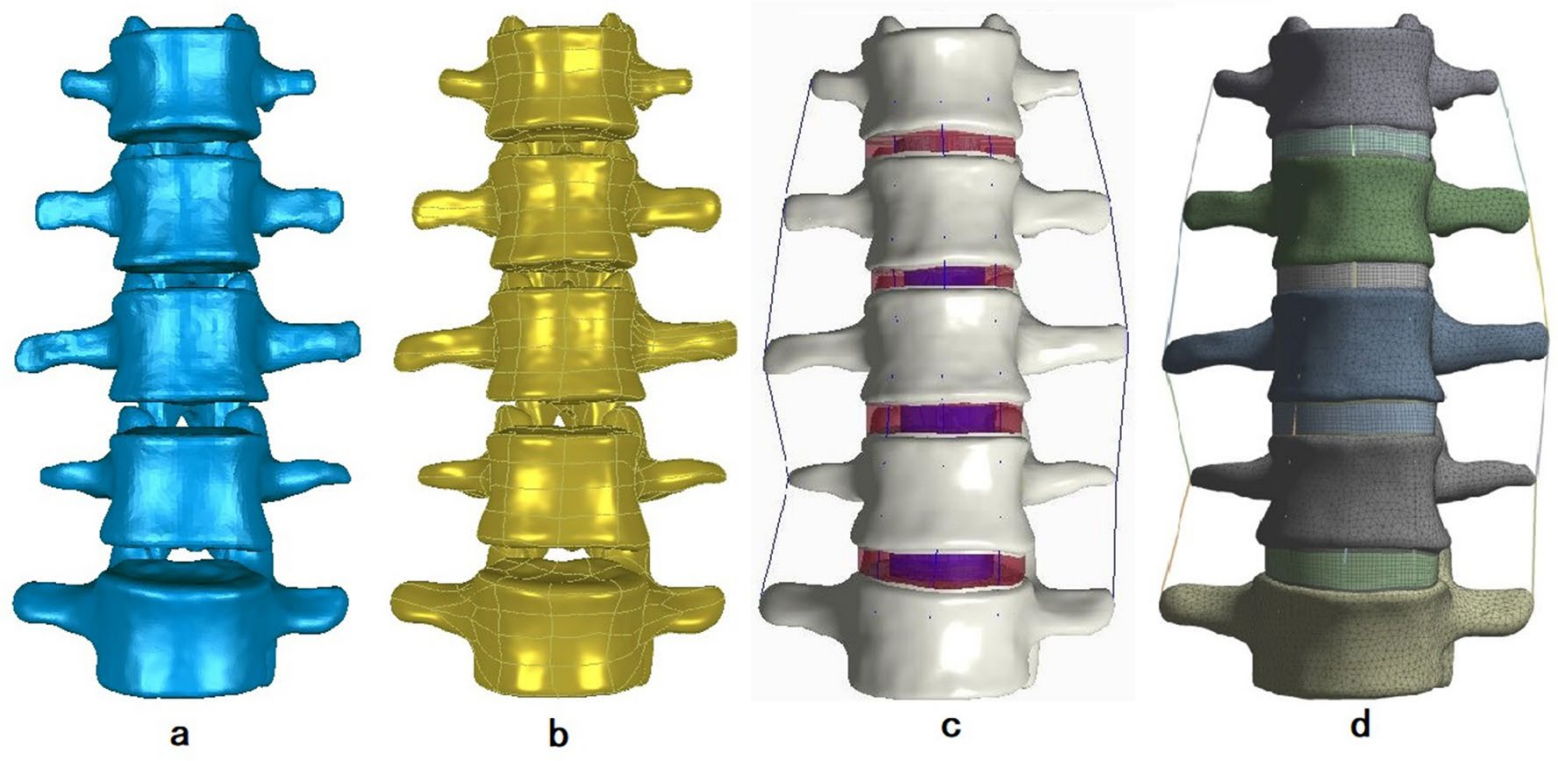
FE models: (**a**) three-dimensional model of the L1 ~ L5 vertebrae, (**b**) the three-dimensional model processed by Geomagic Studio 12.0, (**c**) the three-dimensional model generated by Pro/Engineer5.0, and (**d**) the assembled three-dimensional model for FE analysis.

A healthy adult male volunteer (25 years old, 172 cm, 72 kg) was recruited for a full lumbar thin-layer CT scan (0.625 mm, GE Lightspeed vct-xt 64) after signing the informed consent form. The digital CT images were imported into Mimics 16.0 (Materialise Inc., Leuven, Belgium) to generate a three-dimensional model of the L1–L5 vertebrae (Fig. 6a). The model was saved as an STL file and imported into Geomagic Studio 12.0 (Raindrop Inc., USA) to edit and de-noise the triangular surfaces. The triangular surfaces enveloping each vertebra were ensured to be spatially closed, and finally, the spatially triangular surfaces were fitted into spatially closed NURBS and saved in IGES format (Fig. 6b). Models of the bone, cartilage endplates, annulus fibrosus, nucleus pulposus, and ligaments were constructed using Pro/Engineer5.0 (PTC Inc., Massachusetts, USA) (Fig. 6c). Abaqus (Hibbitt, Karlsson, and Sorensen, Inc., Providence, Rhode Island, USA) was used to define material properties and for FE analysis (Fig. 6d).

The vertebral body consisted of cortical bone and cancellous bone. The thickness of the cortical shell was 1 mm. The thickness of the endplate was set at 0.5 mm^22^. The intervertebral disc was divided into the nucleus pulposus and annulus fibrosus. The nucleus pulposus accounted for 30% ~ 40% of the intervertebral volume^22^ and was modeled as a linearly elastic fluid element. The annulus fibrosus consisted of the annulus ground substance and fibers. Six layers of annulus fibers were embedded into the annulus ground substance at an inclination of ± 30°. The elastic strength of the annulus fibers proportionally increased and varied from the innermost layer (360 MPa) to the outermost layer (550 MPa). The articular cartilage of the facet joints was set as 0.5 mm^23^. A total of seven ligaments were modeled, including the anterior longitudinal ligament, posterior longitudinal ligament, ligamentum flavum, capsular ligament, interspinous ligament, supraspinous ligament, and intertransverse ligament. The element types and material properties used in the FE model were defined according to previous reports^22–25^ and are shown in Table 1.

**Table 1:** Material properties assigned to the FE model^22–25^.

Component	Element type	Young modulus (MPa)	Poisson ratio	Cross-sectional area (mm^2^ )
Cortical bone	C3D4	12,000	0.3	–
Cancellous bone	C3D4	100	0.2	–
Endplate	C3D4	500	0.4	–
Articular cartilage	C3D4	10.4	0.4	–
Nucleus pulpous	C3D8	1	0.49	–
Annulus ground substance	C3D8H	Hyperelastic (C10, 0.2; C01, 0.5)	0.4	–
Annulus fibers	T3D2	Hypoelastic (360–550 MPa)	0.3	–
Anterior longitudinal	T3D2	7.8 (< 12), 20 (> 12%)	0.3	63.7
Posterior longitudinal	T3D2	10 (< 11%), 20 (> 11%)	0.3	20
Ligamentum flavum	T3D2	15 (< 6.2%), 19.5 (> 6.2%)	0.3	30
Capsular	T3D2	7.5 (< 25%), 32.9 (> 25%)	0.3	40
Interspinous	T3D2	10 (< 14%), 11.6 (> 14%)	0.3	70
Supraspinous	T3D2	8.0 (< 20%), 15 (> 20%)	0.3	70
Intertransverse	T3D2	10 (< 18%), 58.7 (> 18%)	0.3	30
Implant (titanium alloy)	C3D4	110,000	0.3	–
Implant (UHMWPE)	C3D4	3000	0.3	–

*UHMWPE* Ultrahigh molecular weight polyethylene.

### FE modeling after implant implantation

**Figure 7:**
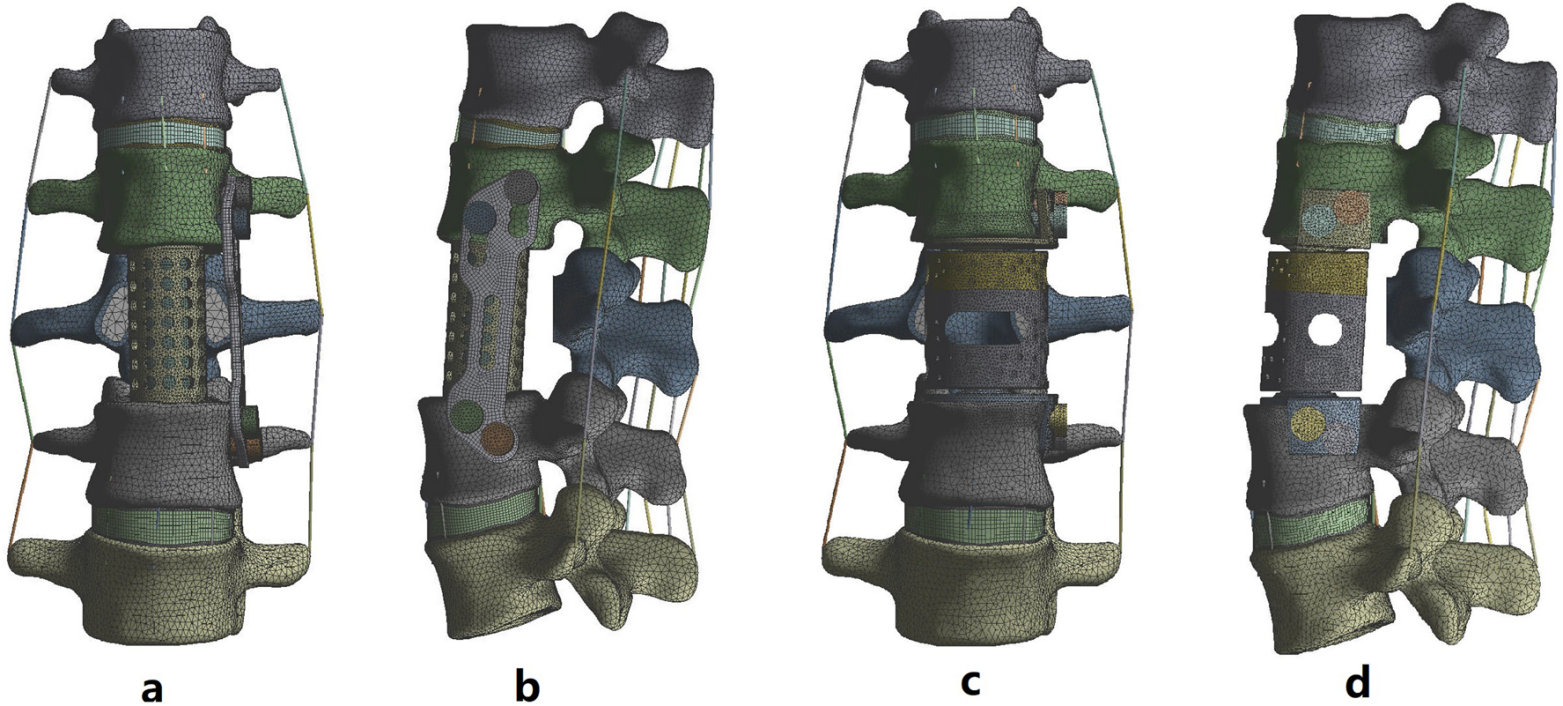
The FE models after prothesis implantation: a front view of the FE model of the fusion group, b lateral view of the FE model of the fusion group, c front view of the FE model of the nonfusion group, and d lateral view of the FE model of the nonfusion group.

The FE models after prothesis implantation are shown in Fig. 7. The modeling processes were as follows. First, the L3 vertebral body and adjacent discs (L2–3 disc, L3–4 disc) were removed. The anterior longitudinal ligament and posterior longitudinal ligament of the surgical site were also removed. However, the posterior vertebral body parts, such as the pedicle and facet joints, were retained. Second, a titanium cage filled with cancellous bone was implanted at the surgical site, a titanium plate and screw were used to assist the lateral fixation, and then the FE model for the fusion group was constructed. The inner diameter of the titanium cage was 20 mm, the height was 46 mm, the thickness was 2 mm, the titanium plate length was 100 mm, the width was 23 mm, and the thickness was 3 mm. The screw was 6.5 mm in diameter and 45 mm in length. Third, the MALV was implanted into the surgical site and fixed with the adjacent vertebral body with four screws to construct the FE model of the nonfusion group. The diameter and length of the screws used were 6.5 mm and 45 mm, respectively. For all FE models above, geometric matching at the prosthesis-endplate interface was achieved using the “Boolean calculation” to remove the portion of the cage and MALV that overlapped with the vertebral body. The effect of the teeth on the titanium cage and MALV surface was minimized by assigning a friction coefficient of 0.2 to the prosthesis-endplate interface^26^. A “tie” constraint was assigned to the interfaces between the screw plate and screw bone to simulate rigid fixation. The contact surface between the ball and socket joint and MALV was smooth.

### Boundary and loading conditions

For all FE models, the lower surface of the L5 vertebra was constrained. An axial preload of 500 N was imposed on the superior surface of L1 to simulate the corresponding physiological compression. Another 10 Nm was applied on L1 to simulate flexion (FLX), extension (EXT), left lateral bending (LLB), right lateral bending (RLB), left rotation (LT) and right rotation (RT). To validate the L1–L5 FE model of the intact group, the segmental ranges of motion (ROMs) (L1–L2, L2–L3, L3–L4 and L4–L5) were compared with the outcomes of previous cadaveric studies. The L1–2, L2–3, L3–4 and L4–5 ROM and maximum von Mises stresses of the disc, endplate and facet joint were measured and compared among the surgical constructs.

### Ethics approval

This study was strictly performed in accordance with the Code of Ethics of the World Medical Association (Declaration of Helsinki) and approved by the Ethics Committee of Zhengzhou University. The volunteer signed the informed consent form and agreed to publication of the study data.
